# ABO blood groups and cardiovascular disease and its risk in continental Africans and people of African ancestry: A systematic review

**DOI:** 10.1371/journal.pone.0333547

**Published:** 2025-10-22

**Authors:** Francis Broni, James Abugri, Samuel Mawuli Adadey, Victor Asoala, Patrick Ansah, Godfred Agongo

**Affiliations:** 1 Navrongo Health Research Centre, Ghana Health Service, Navrongo, Ghana; 2 Department of Biochemistry and Forensic Sciences, School of Chemical and Biochemical Sciences, C. K. Tedam University of Technology and Applied Sciences, Navrongo, Ghana; 3 University of Cape Town, Faculty of Health Sciences, Cape Town, South Africa; Cairo University Kasr Alainy Faculty of Medicine, EGYPT

## Abstract

**Background:**

Cardiovascular disease (CVD) remains a global health challenge and contributes substantially to mortality burden in sub-Saharan Africa (SSA) in particular. Several factors, including particular blood group types in the ABO system, have been associated with CVD risk. However, the direction of the association of ABO blood groups with CVD remains controversial. This review looked at the studies that investigated the association of ABO blood groups and CVD and its risk in SSA and people of African ancestry.

**Methods:**

The review included all observational studies that investigated ABO blood groups and their association with CVD and CVD risk in Africans and people of African descent and were published in English between 1960 and 2023. The data were extracted from Pubmed, Google Scholar, ScienceDirect, Web of Science, Scopus, African Wide and Medline. A total of 24 publications were reviewed following the inclusion criteria. The protocol for this systematic review was registered with PROSPERO (ID#: CRD42023495721).

**Results:**

A total of 24 studies were included in the review with most of them being cross-sectional in design. The mean age of participants was 44 years with an age range of 1–89 years. The most common blood group in SSA was blood group O. The review showed that 11 out of the 24 studies indicated non-O groups association with CVD and CVD risk and 4 studies indicated blood group O association with CVD risk. The most common CVD risk markers studied were body mass index (BMI) and blood pressure (BP). The CVDs investigated were ischaemic disease, intracranial aneurysm, peripheral artery disease and coronary artery disease.

**Conclusion:**

There is no conclusive evidence showing a particular blood group, in the ABO system, being cardioprotective or more susceptible to CVD risk. The varying ABO associations with CVD risk among Africans and African ancestry underscore the importance of targeted and localised interventions aimed at curbing CVD against the backdrop of ABO profiling.

## Introduction

Cardiovascular diseases (CVDs) are major drivers of global mortality. Over 20 million cardiovascular-related deaths occurred in 2021, a figure that accounted for approximately one-third of all global deaths within that period [1]. In contrast to high-income countries that have low CVD burden, that in low-income countries, particularly, in sub-Saharan Africa (SSA), is high partly due to modifiable factors including differences in lifestyle, socioeconomic and environmental factors [2]. Additionally, non-modifiable factors including ABO phenotypes have been linked to CVD risk [3]. However, the influence of these ABO blood group phenotypes on CVD risk in continental Africans and people of African descent remain largely unknown.

The clinical importance of ABO blood types is not only in transfusion medicine and organ transplantation but also in their reported association with other diseases including Cardiovascular disease (CVD) [4,5]. For instance, studies have suggested the association of non-O blood group with coronary heart disease risk, myocardial infarction, peripheral heart disease, cerebral ischaemic stroke and venous thromboembolism [6].

Although, there are reports of the association ABO blood groups, particularly non-O blood groups, with increased rates of cardiovascular events, [5,7,8], the magnitude of the association in different ethnic groups remains controversial. This is partly due to population differences in modifiable and non-modifiable biological risk factors [9]. It is crucial to understand the contribution of genetic polymorphisms including the ABO blood group system to the development of CVDs, particularly in sub-Saharan Africans and people of African descent. This may contribute to improving risk stratification and enhance our ability to tailor public health interventions aimed at reducing the burden of CVDs in African populations. This study reviewed literature on the contribution of ABO blood groups to CVD risk and the development of CVDs in individuals of African ancestry with the aim of contributing to global health knowledge on CVDs and promoting the importance of personalized medicine to addressing health disparities in Africa.

## Methods

### Database search strategy

We reviewed the literature to investigate the association between ABO blood group and CVD and its risk markers among people of African ancestry. The protocol for this systematic review (S1 Appendix) was registered with PROSPERO [10] (ID#: CRD42023495721). The systematic review was conducted using the Preferred Reporting Items for Systematic Reviews and Meta-Analysis (PRISMA) framework [11]. The PRISMA checklist (S1 Table) is included in the supplementary material. To prevent duplication of systematic reviews based on the topics addressed in this study, the Cochrane Library (https://www.cochranelibrary.com), PROSPERO (the international prospective register of systematic review, https://www.crd.york. ac.uk/prospero), and google scholar were used to check for similar reviews.

PubMed, Google Scholar, and ScienceDirect, Web of Science, Scopus, African Wide and Medline were searched for relevant papers published between 1960 and 2023. The search on each database was conducted from 4^th^ to 31 January 2024 by two independent reviewers. Articles written in English were considered for the review.

The keywords ‘ABO blood group’, ‘ABO blood genotypes’ ‘cardiovascular risk markers’, ‘sub-Sahara Africa’, ‘continental Africans’, ‘African ancestry’ and ‘people of African descent’ were used to develop the search term as follow; (“ABO blood groups” OR “ABO blood genotypes” OR) AND (“Cardiovascular risk” OR “Cardiovascular risk markers”) AND (“Sub-Saharan African”) OR (“Continental Africans”) AND (“People of African ancestry”) OR ((“People of African descent”).

### Inclusion and exclusion criteria

We included studies published in the English language that focused on identifying ABO blood groups or ABO alleles for CVD and its risk markers. The following inclusion and exclusion criteria were used to screen the records retrieved.

Observational studies (cross-sectional, cohort, case-control, case series, and case reports) reporting ABO blood group or gene variants associated with CVD and its risk markers in SSA and people of African descent were included in this review.

Studies that did not report on ABO blood groups and CVD risk were excluded from this review. Additionally, populations of non-continental Africa and non-African descent, qualitative studies, editorials, letters to editors, reviews and communications were excluded from this review.

### Data collection processes

Published studies were identified through database searching, against the selection criteria and screened by titles and abstracts. Full articles were obtained for studies that met the inclusion criteria (full details in S2 Table) and where studies were excluded, two independent persons recorded reasons for exclusion (S3 Table). Screening of the full text articles, and selected studies in accordance with the inclusion criteria were conducted by two independent reviews, checked by BF, GA, with final inclusion by consensus.

### Data extraction and evaluation

Data was extracted from the included studies in the form of a summary table (S2 Table). All authors screened the extracted data and ambiguities were resolved through discussion (S4 Table). Data was evaluated by following the guidelines defined by Sagoo et al., [12] i.e., assessment by risk of bias in individual studies and across studies case/trait adjustment definition, population stratification, reporting of methods used (sample size of a study population, phenotyping and genotyping method and its reliability/accuracy, validation of results, statistical analyses). This reduced publication bias within this systematic review (avoiding risk of bias caused by selective reporting).

The outcome variables used in the presentation of results were CVD and CVD risk, the independent variables were ABO types, and the effect measures were risk ratios, odds ratios, β values, and χ^2^ values.

### Risk of bias assessment

To evaluate the risk of bias, a modified version of the Newcastle-Ottawa Scale (NOS) for non-randomized studies in meta-analyses was employed [13]. Assessment guidelines were drawn from the manual of the risk of bias tool. Given that most of the included publications lacked detailed reporting on bias assessment and participant selection methods, a specific subset of questions from the risk of bias tool was applied uniformly across studies. Additionally, each article was evaluated and ranked based on the quality and reporting standards of the biological and biomedical data presented (see S5 Table).

## Results

**Fig 1:**
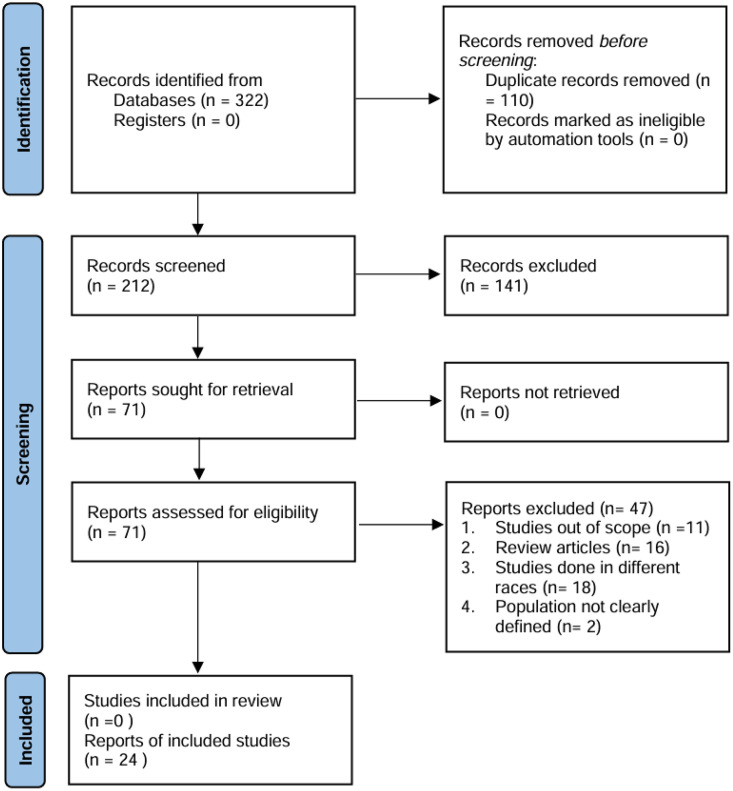
PRISMA diagramme for search and strategies for identification of included reports.

The number of records identified from the database was 322. After duplicate records were removed and screening done to exclude all other records that did not meet the inclusion criteria, a total of 24 records were retained for data extraction (Fig 1).

**Fig 2:**
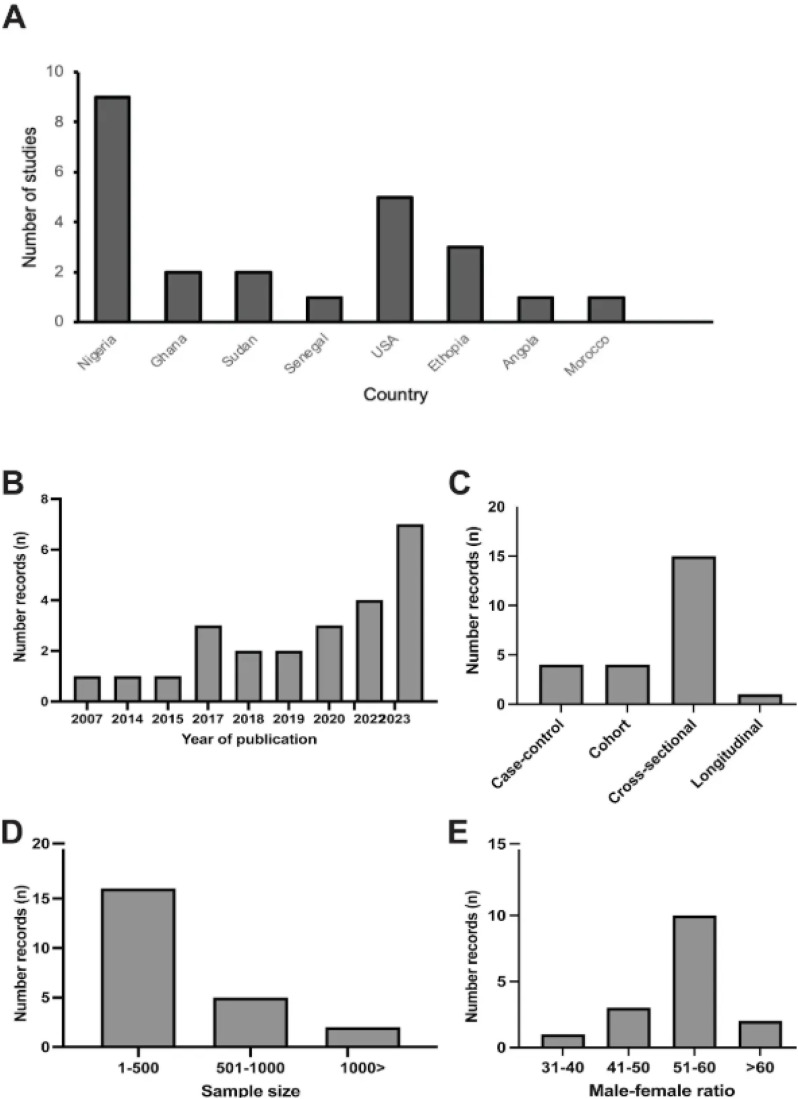
Characteristics of publications included in this study. **(A)** Country representation of publications retrieved. **(B)** The year of publication for the selected records. **(C)** Study design used by the various studies. **(D)** Sample size of the selected records. **(E)** Gender distribution of study participants.

The earliest study among these 24 records was conducted in 2007, while the year 2023 recorded the highest number of studies (Fig 2B). Most ABO blood group and cardiovascular risk marker studies (12/24) were conducted in West African countries with the highest frequency from Nigeria. There were few studies recorded from South Africa, East Africa and North Africa. Five of the studies were conducted in the United State of America (USA) (Fig 2A).

A review of the study designs used suggested that cross sectional studies were the most common study designs (Fig 2C). Most of the records reviewed had less than 500 participants and male to female ratio of the studies included was almost the same number (Fig 2D and 2E) respectively. Biochemical, anthropometric, and slide and tube agglutination techniques were used to determine cardiovascular risk markers and associated ABO blood group phenotypes.

### Distribution of ABO blood group phenotypes

The pool mean age of the participants from the records included was 44 years. The age range of the participants was 1–89 years.

**Fig 3:**
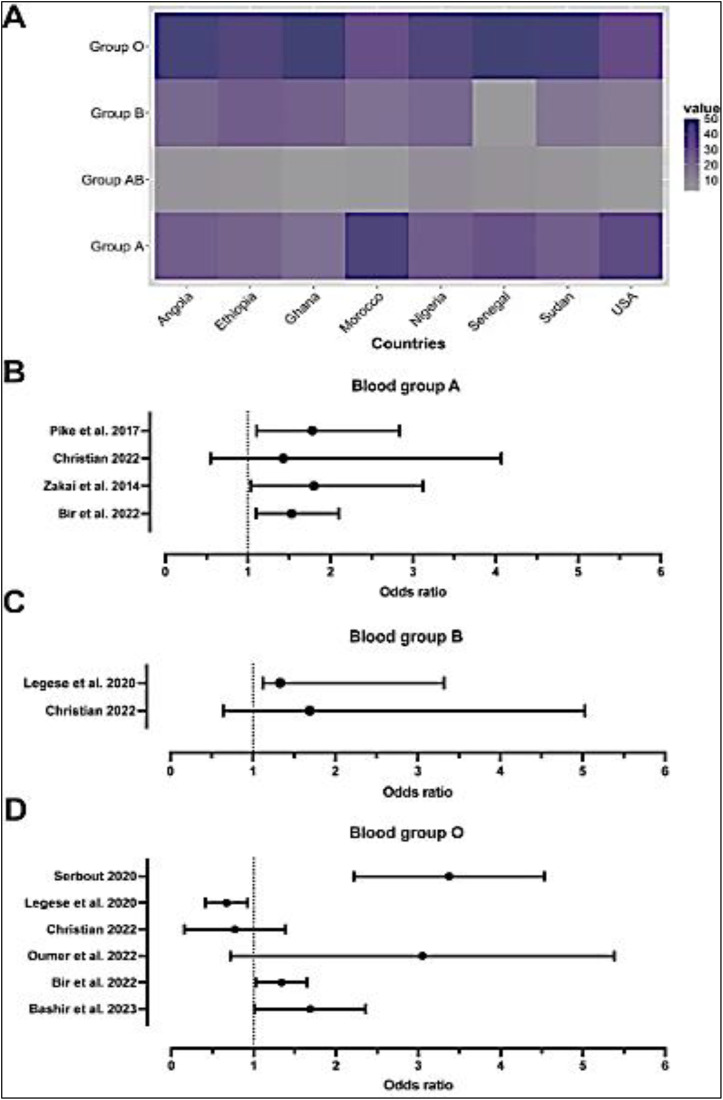
Distribution of ABO blood phenotypes and cardiovascular disease risk. (A) Average proportion of blood groups recorded. (B) Forest plot of odds ratio, a measure of ABO blood group association CVDs.

The most common ABO blood phenotype in the study populations was blood group O except for the USA and Morocco that had high frequencies of blood group A (Fig 3A). The ABO blood phenotypes were determined chiefly (15/24) by agglutination methods, with slide agglutination being the commonest method (9/15). Three studies used genetic methods based on single nucleotide polymorphism (SNP) genotyping to infer the ABO blood group types.

### ABO blood group and CVD and CVD risk markers

The most common CVD risk markers studied were Body Mass Index (BMI), and Blood Pressure (BP). Majority of the studies investigated at least two or more risk markers. Most of the studies employed biochemical and anthropometric methods to investigate the CVD risk markers in the population (Table 1). Additionally, CVDs including Ischaemic disease, intracranial aneurysm, peripheral artery disease and coronary artery disease were associated with ABO blood groups (Table 1).

**Table 1:** CVD and its risk markers and associated ABO blood groups.

CVD and CVD Risk Factors	Blood group Associated	Country	Reference
BMI, Lipidemia	Antherogenic Index (AI) were significantly associated with ABO blood group phenotypes (χ2 = 20.160, p = 0.003). The study lacks specific ABO phenotype relationships.	Nigeria	[14]
BP, BMI	Blood group B is associated with hypertension (OR= 0.091, 95% CI = 0.010–0.791; p = 0.030)	Ghana	[15]
BMI, Lipidemia	Blood group O is associated with high BMI (Coefficient = 0.68; p = 0.035) Similarly, blood group O together with other factors were associated with obesity (AOR = 1.60, 95% CI = 1.06–2.04)	Sudan	[16]
BP, BMI, Pulse rate	ABO blood groups were not associated with increased BP, BMI and increased pulse rate.	Nigeria	[17]
BP, BMI, CRP	C -reactive protein (CRP) was significantly lower in blood group O (4.19 ± 3.60 mg/L, p = 0.025) participants compared with blood group AB, B and A (8.11 ± 4.04 mg/L, 6.31 ± 4.32 mg/L, 6.57 ± 4.31 mg/L) respectively.	Nigeria	[18]
BMI, Lipidemia, BP, DM	Male subjects of blood group O (51.7%) were associated with an increased risk for CVD (χ2 = 6.213; p = 0.045) .	Nigeria.	[19]
BMI, BP, DM, Smoking	Diabetes and hypertension were more common in non-A blood groups (95% CI = 0.015–0.60, p = 0.015 and 95% CI = 0.08–0.71, p = 0.014 respectively) compared with A blood group. The diagnosis of ischemic disease (ID) was higher in patients with blood group A (61.2%) than in other blood groups, and the diagnosis of non-ischemic disease (NID) was higher in patients with blood group O (73.6%) compared to other groups.	Senegal	[20]
BMI, BP, DM, Smoking	ABO phenotype is associated primarily with height and the observed associations with lipids, lipoproteins and blood pressure are by virtue of their correlation to height.	USA	(Borecki et al., 1985)
DM, BMI, VTE	Non-O blood type was associated with risk of venous thromboembolism (VTE) with OR of 1.65 (95% CI = 1.31–2.05). Each non-O blood type had a higher risk of VTE compared with the O blood type; the age-adjusted ORs were 1.65 (95% CI, 1.29–2.10) for type A, 1.74 (95% CI, 1.25–2.44) for type B, and 1.34 (95% CI, 0.77–2.32) for type AB.	USA	[21]
Lipidemia	CVD risk was higher in non-O blood groups (AB, A, B,) with females having the highest risk. CVD risk profile of the blood groups in the male subjects using low density lipoprotein cholesterol (LDL-C) shows that the risk of CVD was highest in subjects of blood group A (41.67%), followed by blood group AB (33.33%), blood group O (30.56%) and blood group B (20.00%). In the females, the risk profile for CVD increased in the order of blood group AB (67.67%), blood group A (62.50%), blood group O (41.67%) and blood group B (35.71%).	Nigeria	[22]
BP, DM, smoking, BMI, Lipidemia, IA	Smokers with blood group O have a high chance of intracranial aneurysms (IA). Similarly high BMI patients with blood group A have high odds of developing IA	USA	[23]
BP	Uncontrolled blood pressure was higher among blood group O individuals.	Ethiopia	[24]
DM, BP, stroke	Blood group AB was associated with stroke risk independent of conventional risk factors.	USA	[9]
DM	Blood groups A, B and O contribute significantly to developing diabetes mellitus of odd ratio 0.065, 0.059 and 0.037 respectively at 95% CI.	Ethiopia	[25]
BMI, DM	Non-O (A, B, AB) blood group individuals have higher odds of developing diabetes mellitus compared to O blood group individuals who have better glycemic control. The fasting blood sugar (FBS), two hour postprandial (2HPP) and glycated haemoglobin (HBAIC) levels were increased for subjects with non-0 blood groups (A, B, AB) compared to subjects of blood group O, though, not statistically significant (p = 0658, 0.592, 0.358) respectively.	Nigeria.	[26]
CHD	Coronary heart disease was most frequent among patients with non-O blood group 280 (56%) compared to O blood group 220 (44%).	Sudan	[27]
BMI	Overweight and obesity were not associated with ABO blood groups	Ghana	[28]
DM	Majority (46%) of the patients were blood group O	Angola	[29]
BMI, BP, Lipidemia, Ankle brachial index (ABI)	Blood type A was associated with lower ABI in African Americans (p = 0.014). Similarly, there is increased odds of prevalence between blood A and peripheral artery disease	USA	[30]
BMI, BP, Lipidemia, DM	Blood group A individuals have 2.35 times more at risk to hypertensive compared to other blood groups.	Ethiopia	[31]
ID, Non Ischemic Disease (NID), Lipidemia	Ischemic disease (ID) was higher in patients with blood group O (63.3%) (95% CI = 2.26–4.57, p < 0.02) than in other blood groups.	Morocco	[32]

Most of the studies have suggested that Non-O groups of the ABO blood phenotype were associated with CVD risk markers in Africans and African ancestry (Fig 3B).

## Discussion

Our review of literature on continental Africans and people of African ancestry indicates that the most common blood group is O and analysis of the results implicated ABO blood groups in CVD and/or CVD risk. With the exception of data from 4 out of 24 studies [16,19,23,24] which showed blood group O association with CVD risk, and a study [14] which showed no difference in association among the phenotypes, 11 out of the 24 reviewed studies indicated that non-O groups are more frequently associated with CVD or CVD risk. It is worth noting that 8 of the records retrieved did not clearly associate ABO blood group with CVD or CVD risk. This general observation of non-O group association with CVD risk has been supported by findings of several other studies among non-Africans [7,33–35].

The evolution of the ABO blood group system has been explained differently. While group O is suggested as being the original blood group and A and B arising from mutations, another theory suggests group O arising from the inactivation of A1 glycosyltransferase gene due to selection pressure and resistance to disease, particularly malaria [35,36]. The second theory probably explains why blood non-O groups are observed to be more associated with CVD and CVD risk in Africans and African ancestry. The observed higher proportion of blood group O than the non-O groups in Africans and African ancestry and the CVD risk association with non-O groups could be due to the selection advantage of group O over the non-O groups.

Our review further demonstrates that different ABO phenotypes are associated with different CVD risk markers among different populations. This suggests the diversity among Africans in the association of their blood group phenotypes with CVD risk. Studies have demonstrated the genetic diversity of the African populations [37–39] and this may contribute to observed differences in phenotype associations, and suggesting the need for personalized medicine.

Some of the studies have demonstrated sex differences in the association of the ABO phenotypes with CVD risk. In the study by Anioke et al. [16] group O in males was more associated with CVD risk while Bartimaeus et al reported association of AB phenotypes with CVD risk among females. The study by Bir et al. [40] in which smokers with blood group O were associated with greater risk of developing intracranial aneurysms (IA) suggests the modulation of environmental factors in the influence of ABO blood group in CVD risk.

Thus, the review of the literature suggests that blood group O is the dominant group among Africans and people of African ancestry, and that the non-O groups are more commonly associated with CVD risk than the O group. Additionally, several factors including sex, environmental exposures and population architecture may influence the contribution of ABO blood groups to CVD risk. This underscores the need for targeted public health interventions aimed at curbing CVD within the context of risk profiling using ABO blood group system with the consideration of sex, environmental factors and genetic diversity in African and African ancestry populations.

The strength of our review lies in its coverage of varied African populations which are diverse. Our review is the first to comprehensively evaluate existing literature on ABO blood groups and their influence on CVD risk in continental Africans and people of African ancestry. The review shows that non-modifiable (sex, age) and modifiable factors (environmental exposures) may modify the contribution of ABO blood groups to CVD risk. However, our review is not without limitations. Different methodologies have been used in the different studies making it difficult to compare findings. Most of the studies that have been reviewed are cross sectional and they suggest associations of ABO blood groups with CVD risk and not causality. Additionally, most of the studies investigated association of ABO blood groups with CVD risk markers but not with CVD. Nonetheless, our review has contributed to the general understanding of the most prevalent ABO blood groups among Africans and people of African ancestry and the association of these blood groups with CVD and its risk.

## Conclusion and recommendation

In conclusion, our review showed that blood group O was the most common in the ABO system among Africans and people of African descent. There are varied findings regarding the association of ABO blood groups with CVD risk and that these findings may be modulated by other factors including sex and environmental exposures among Africans and people of African descent. Though there is no conclusive evidence showing a particular blood group, in the ABO system, being predominantly cardioprotective or susceptible to CVD risk among Africans and people of African descent, the effect of the ABO system on CVD risk may be population or environment specific. Therefore, our review shows the usefulness of the input of ABO blood group phenotypes in CVD risk profiling within the context of environmental and demographic variables, demonstrating the need for tailored and targeted public health strategies aimed at reducing CVD burden.

We recommend that longitudinal studies involving large cohorts be conducted within SSA to establish blood groups that may be susceptible to CVD.

## Supporting information

S1 Table Prisma checklist. (DOCX)

S1 Appendix The study protocol. (DOCX)

S2 Table Studies that met the inclusion criteria. (XLSX)

S3 Table Records excluded from the review with reasons. (XLSX)

S4 Table Agreements between two independent data extractors confirming the eligibility of the study for review. (XLSX)

S5 Table Risk of bias assessment. (XLSX)
