# Supplementary material for: ABO blood groups and cardiovascular disease and its risk in continental Africans and people of African ancestry: A systematic review
Source: PLoS One. 2025 Oct 22;20(10):e0333547. doi: 10.1371/journal.pone.0333547 (PMC12543130; doi:10.1371/journal.pone.0333547)
Supplement: S1 Appendix — (DOCX) [file pone.0333547.s002.docx]

**Systematic Review Protocol**

**Title**: ABO genotypes and cardiovascular disease risk in continental Africans and people of Africa Ancestry: A systematic review

Citation: Francis Broni, James Abugri, Samuel Mawuli Adadey, Victor Asoala, Patrick Ansah, Godfred Agongo. ABO genotypes and cardiovascular disease risk in continental Africans and people of Africa Ancestry: A systematic review.

**Review Question**

What are the commonly reported cardiovascular risk markers in people of Africa decent?

What is the association between ABO blood groups and cardiovascular risk markers?

**Searches**

The search will be conducted with the outlined strategy:

1. Databases: PubMed, Google Scholar, and Science direct, Web of Science, Scopes, African Wide and Medline.

2. Date: The search on each database will be conducted from 4^th^ to 31 January 2024 by two independent reviewers.

3. Language: Articles written in English will be considered for the review.

4. Keywords: (“ABO blood groups” OR “ABO blood genotypes” OR) AND (“Cardiovascular risk” OR “Cardiovascular risk markers”) AND ("Sub-Saharan African”) OR (“Continental Africans”) AND (“People of African ancestry”) OR ((“People of African descent”)

**Types of study to be included**

We will include studies that are focused on identifying ABO blood group or ABO alleles for cardiovascular risk markers. The following are inclusion and exclusion criteria;

1- Inclusion criteria: Observational studies (cross-sectional, cohort, case-control, case series, and case reports) reporting ABO blood group or gene variants associated with cardiovascular disease risk markers.

2- Exclusion criteria: populations of non-continental Africa and non-African descent, qualitative studies, editorials, letters to editors, reviews and communications.

**Condition or domain being studied**

ABO blood group is associated with several diseases. People with blood group O have a lower risk of cardiovascular disease (CVD), including myocardial infarction (MI), peripheral vascular disease, cerebral ischemic events, and venous thromboembolism, as well as of digestive system neoplasms (gastric and pancreatic cancer) and ovarian cancer, than people with other blood groups. Findings from this study will contribute to precision medicine within the field of CVD in Sub-Saharan Africa and people of African descent.

**Participants/population**

Individuals with or without cardiovascular disease risk in Sub-Saharan Africa or people of African descent.

**Intervention(s), exposure(s)**

(1) lifestyle changes e.g. change in diet and smoking habits, (2) Exercise, (3) controlled hypertension and diabetes (4) weight loss

**Comparator (s)/Control**

Control participants with low cardiovascular risks and no diagnosis of cardiovascular diseases

**Context**

All types of studies describing cardiovascular risk markers and ABO blood group or genotypes.

**Main outcome(s)**

The distribution of ABO blood types in the sub-region would be evaluated.

The association of ABO blood types to the development of cardiovascular disease risk will be examined.

The relative risk and the contribution of ABO blood group to the development of cardiovascular diseases will be determined.

**Measures of effect**

The measure of effect will be the relative risk of ABO genotypes and cardiovascular risk markers.

**Additional outcome(s)**

prevalence, and frequencies

**Measures of effect**

Effect size for relative risks will be calculated.

**Study selection**

A blinded screening will be done by the two reviewers using the titles and abstracts. The independent search will be downloaded into zotero referencing software and duplicates will be removed. The screening results will be compared and merged.

**Data extraction**

The data extraction will be conducted independently by the two reviewers and compared to remove any form of bias.

The following will be extracted from the documents: 1) the last name of the first author, 2) date of publication, 3) location, 4) range, mean, and median age, 5) sample size, 6) sampling method, 7) methods of ABO blood grouping/ genotyping, 8) Frequency and percentage of each blood group category or ABO genotype

The data extracted will be manually captured on to Microsoft Excel sheets and analyzed using STATA version 16. A third person (SMA) who is an expert in the field would be consulted in times of disagreements between the individual judgements of the reviewers during the screening and data extraction process.

**Risk of bias (quality) assessment**

To avoid any form bias, two reviewers will independently synthesize the data and assess the quality of the documents included. The National Institute of Health (NIH) quality assessment tools will be used to assess the quality of the data in the included studies. The tool is preferable because it is more comprehensive and thus enables exhaustive assessment of the quality of the included studies. The overall quality of included studies will be rated as good, fair and poor.

**Strategy for data synthesis**

The study selection will be clearly illustrated using a flow diagram with the reasons for full-text exclusion described. The reviewers will clearly outline how the studies included synthesized their findings by reporting on the following.

1. All participants with cardiovascular risk will be considered and their and country of origin will be captured.
2. What ABO group or genes are associated? How many with particular cardiovascular risk marker identified with ABO group or gene?

The data extracted will be analyzed using the STATA 16. To estimate the prevalence of the various cardiovascular risk markers, a fraction of the number of participants with the risk marker and the total number of participants will be computed and reported at the country. Quantitative data obtained from the studies will be presented in tables (descriptive summary tables), charts, and plots where appropriate.

**Analysis of subgroups or subsets**

Not Applicable

**Contact details for further information**

Francis Broni

Francisbroni75@gmail.com

**Organisational affiliation of the review**

C. K. Tedam University of Technology and Applied Sciences

**Review team members and their organisational affiliations**

Mr. Francis Broni, C. K. Tedam University of Technology and Applied Sciences; Navrongo Health Research Centre

Prof. James Abugri, C. K. Tedam University of Technology and Applied Sciences

Dr. Samuel Mawuli Adadey, University of Cape Town.

Dr. Victor Asoala, Navrongo Health Research Centre

Dr. Patrick Ansah. Navrongo Health Research Centre

Dr. Godfred Agongo, C. K. Tedam University of Technology and Applied Sciences

**Type and method of review**

Systematic review.

**Anticipated or actual start date**

12 December 2023

**Anticipated completion date**

30 April 2024

**Funding sources/sponsors**

Not Applicable

**Conflicts of interest**

None

**Language**

English

**Country**

Ghana

**Stage of review**

Review Ongoing

**Subject index terms status**

**Subject index terms**

**Date of registration in PROSPERO**

Yet to register

**Date of first submission**

Not Applicable

**Stage of review at time of this submission**

| **Stage** | **Started** | **Completed** |
| --- | --- | --- |
| Preliminary searches | Yes | No |
| Piloting of the study selection process | No | No |
| Formal screening of search results against eligibility criteria | No | No |
| Data Extraction | No | No |
| Risk of bias (quality) assessment | No | No |
| Data analysis | No | No |
